# Ion-pumping microbial rhodopsin protein classification by machine learning approach

**DOI:** 10.1186/s12859-023-05138-x

**Published:** 2023-01-27

**Authors:** Muthu Krishnan Selvaraj, Anamika Thakur, Manoj Kumar, Anil Kumar Pinnaka, Chander Raman Suri, Busi Siddhardha, Senthil Prasad Elumalai

**Affiliations:** 1 MTCC-Microbial Type Culture Collection and Gene Bank, Institute of Microbial Technology, Council of Scientific and Industrial Research (CSIR-IMTECH), Chandigarh, 160036 India; 2 Virology Unit and Bioinformatics Centre, Institute of Microbial Technology, Council of Scientific and Industrial Research (CSIR-IMTECH), Chandigarh, 160036 India; 3 Biosensor Department, Institute of Microbial Technology, Council of Scientific and Industrial Research (CSIR-IMTECH), Chandigarh, 160036 India; 4 Department of Microbiology, School of Life Sciences, Pondicherry University, Puducherry, 605014 India; 5 Biochemical Engineering Research and Process Development Centre, Institute of Microbial Technology, Council of Scientific and Industrial Research (CSIR-IMTECH), Chandigarh, 160036 India

**Keywords:** Microbial rhodopsins, Actinorhodopsin, Bacteriorhodopsin, Halorhodopsin, Proteorhodopsin, Sensory rhodopsin, Xanthorhodopsin, Support vector machine, SVM, Protein prediction

## Abstract

**Background:**

Rhodopsin is a seven-transmembrane protein covalently linked with retinal chromophore that absorbs photons for energy conversion and intracellular signaling in eukaryotes, bacteria, and archaea. Haloarchaeal rhodopsins are Type-I microbial rhodopsin that elicits various light-driven functions like proton pumping, chloride pumping and Phototaxis behaviour. The industrial application of Ion-pumping Haloarchaeal rhodopsins is limited by the lack of full-length rhodopsin sequence-based classifications, which play an important role in Ion-pumping activity. The well-studied *Haloarchaeal* rhodopsin is a proton-pumping bacteriorhodopsin that shows promising applications in optogenetics, biosensitized solar cells, security ink, data storage, artificial retinal implant and biohydrogen generation. As a result, a low-cost computational approach is required to identify Ion-pumping *Haloarchaeal* rhodopsin sequences and its subtype.

**Results:**

This study uses a support vector machine (SVM) technique to identify these ion-pumping *Haloarchaeal* rhodopsin proteins. The haloarchaeal ion pumping rhodopsins viz., bacteriorhodopsin, halorhodopsin, xanthorhodopsin, sensoryrhodopsin and marine prokaryotic Ion-pumping rhodopsins like actinorhodopsin, proteorhodopsin have been utilized to develop the methods that accurately identified the ion pumping haloarchaeal and other type I microbial rhodopsins. We achieved overall maximum accuracy of 97.78%, 97.84% and 97.60%, respectively, for amino acid composition, dipeptide composition and hybrid approach on tenfold cross validation using SVM. Predictive models for each class of rhodopsin performed equally well on an independent data set. In addition to this, similar results were achieved using another machine learning technique namely random forest. Simultaneously predictive models performed equally well during five-fold cross validation. Apart from this study, we also tested the own, blank, BLAST dataset and annotated whole-genome rhodopsin sequences of PWS haloarchaeal isolates in the developed methods. The developed web server (https://bioinfo.imtech.res.in/servers/rhodopred) can identify the Ion Pumping Haloarchaeal rhodopsin proteins and their subtypes. We expect this web tool would be useful for rhodopsin researchers.

**Conclusion:**

The overall performance of the developed method results show that it accurately identifies the Ionpumping *Haloarchaeal* rhodopsin and their subtypes using known and unknown microbial rhodopsin sequences. We expect that this study would be useful for optogenetics, molecular biologists and rhodopsin researchers.

**Supplementary Information:**

The online version contains supplementary material available at 10.1186/s12859-023-05138-x.

## Background

Rhodopsin is present in a wide range of organisms, from vertebrates to bacteria. Rhodopsin consists of seven retinal chromophore-associated transmembrane helix proteins belonging to the superfamily of GPCRs that act as photoreceptors [1, 2]. Based on the seven transmembrane topology, the rhodopsins are classified into two groups: type-I Microbial Rhodopsin and type-II animal Rhodopsin. Type-I microbial rhodopsins consist of seven transmembrane domain that is covalently associated with retinal chromophore functions like proton pumping, chloride pumping, and phototaxis behaviour. The type-I microbial rhodopsins used in this study, such as actinorhodopsin, bacteriorhodopsin, proteorhodopsin, xanthorhodopsin, belong to the proton pumping type-I microbial rhodopsins family. Halorhodopsin and sensory rhodopsin functions like non-proton-pumping type-I Microbial rhodopsin, such as chloride pumps and photoreceptors. Bacteriorhodopsin is the first microbial rhodopsin to be isolated and well-characterized from the *Halobacterium salinarium* in the 1970s by Oesterhelt and stockineus group [3]. The Light driven proton pump bacteriorhodopsin extensively used in several biophotonics and Bioelectronics applications [4]. Proton pump proteorhodopsins were first discovered during environmental sequencing of pacific coastal waters and deep ocean samples. Proteorhodopsins are the largest subfamily of type-I rhodopsins. 13% of proteorhodopsins harboring bacterial cells live in the photic zone of oceanic marine samples. Proteorhodopsin is the largest type-I microbial rhodopsin subfamily among marine proteobacteria [5, 6]. Xanthorhodopsin, originally found in *Salinibacter ruber* binds to salinixanthin-like carotenoids that bind specifically to the rhodopsin protein. These carotenoids contain a retinal chromophore that absorbs light and transfers energy to the rhodopsin protein in hypersaline *Haloarchaea*. The light-driven proton pump was transformed into halorhodopsin due to Asp 85 single mutation which acts as proton acceptor [7, 8]. ActR gene lineage is also the one of the globally abundant Type-I microbial rhodopsin gene. Actinorhodopsin was first reported in the freshwater lakes in the actinobacteria. Subsequent findings suggested that actinorhodopsin is present abundantly in the terrestrial and ocean environments [9, 10]. Light-modulated swimming behavior is a well-known feature of sensory rhodopsins I. Takahashi and colleagues suggested the existence of a second sensory photoregulatory receptor, rhodopsin II, present in *Halobacterium salinarium* for their repellent response under highly aerobic conditions and showed slow photocyclic processes [11, 12]. Many computational methods have been developed to identify or predict the proteins and their functions, based on protein structure, DNA binding sites, glycosylation sites, subcellular localization and hybridization-based prediction methods [13–15]. Recently a research group Jeanthon from France has developed a MicRhoDE is a comprehensive database that categorize the different types of microbial rhodopsins and their taxonomy classification [16]. Research group Kandori and Takeuchi from Japan developed a machine learning approach to predict the light absorption properties of microbial rhodopsin [17]. Classification and prediction of GPCRs based on amino acid sequences have been reported using a three-layer approach [18, 19]. The isolation of rhodopsin proteins from wild type Haloarchaeal culture is laborious, expensive involves lengthy procedures. The well studied bacteriorhodopsin protein from Haloarchaeal strains has a wide range of applications in Biophotonics and bioelectronic applications. Therefore, it is necessary to identify the bacterial rhodopsin proteins that express in their wild type as well as additional microbial rhodopsin proteins with restricted expression at the mg/l expression level. The full length bacteriorhodopsin sequence also plays a crucial role in the ion pumping activity of recombinant bacteriorhodopsin, which helps to facilitate the development of recombinant bacteriorhodopsin. Full length microbial rhodopsin expressed at high levels is useful for finding new rhodopsin proteins with ion pumping capabilities through crystallography studies.

Currently, GPCR is the only rhodopsin superfamily that has been studied in detail using support vector machine learning by multiple research groups [20]. As per our knowledge, there were no reports on the classification of microbial rhodopsin proteins by support vector machine (SVM). Here, we have developed a method for identification of Ion pumping *Haloarchaeal* rhodopsin using amino acid composition (AAC), dipeptide composition (DPC), and hybrid models. Support vector machine is a supervised machine learning method that has been used in various bioinformatics studies to classify GPCR, proteins of oxygen-binding, plasminogen activators and evolutionary relationship of receptor-associated proteins (RAPs) [21–23]. SVM is a powerful predictor tool that has been extended to many clinical investigations beyond protein studies [15]. It is well-established that sequence-based SVM statistical predictors for biological systems are susceptible to the following rules: (a) Data set construction, (b) Program the biological sequence in mathematical terms (c) Develop a robust algorithm (d) Perform cross-validation to evaluate prediction accuracy (e) Run the algorithm using the server user-friendly online web [24]. SVM models have been created for bacteriorhodopsin, actinorhodopsin, xanthorhodopsin, proteorhodopsin, sensory rhodopsin, and halorhodopsin. To run the SVM to generate models, a sequence of subclasses is labelled as positive and negative every other classes are labelled as negative [25]. When creating classification models, it is repeated for all classes. Each of the five SVM models was developed by employing a fivefold cross validation procedure that is identical in both techniques. To recognise the classes depicted in the prediction score graphs, Each and every sequence in the dataset was analyzed using recently constructed models. Haloarchaeal rhodopsin proteins and subtypes were also identified using the blind dataset. The accuracy (ACC), sensitivity (SN), and specificity (SP) of the prediction results were compared with in the classes [26]. SVM classifiers integrated with rhodopred webserver correctly identified the subtype of Ion pumping Haloarchaeal rhodopsin and experimentally validated whole-genome Haloarchaeal rhodopsin sequences extracted from NCBI and Haloweb Genome web databases (https://www.haloweb.org/) [27]. This SVM method focuses on the prediction and analysis of various ion-pumping *Haloarchaea* rhodopsins of recently isolated *Haloarchaeal* strains whole genome data available in the NCBI (https://www.ncbi.nlm.nih.gov/genome/) database using the Rhodopred web server. Among the Type-I Ion pumping Microbial rhodpsins the sensory rhodopsins were out grouped from the chloride pumping rhodopsins were different from Ion Pumping rhodopsin amino acid sequences.

The developed SVM models suggest that full-length rhodopsin sequences are responsible for Ion pumping properties of type-I microbial rhodopsin, which would be helpful in heterologous protein expression and optogenetics studies [28].

## Methods

The present method classifies the ion pumping type-I microbial rhodopsin by combining the amino acid composition (AAC) and the dipeptide composition (DPC) in order to get a higher level of precision. These predictive models were developed to compare the type-I microbial rhodopsin amino acid sequences using -5-fold and 10-fold cross-validation methods. Amino acid composition (AAC), dipepide composition (DPC), and hybrid (HYB) approach were used to build the predictive models. The known and experimentally verified rhodopsin sequences extracted from NCBI, and Haloweb genome database were given as input in the rhodopred web server. Based on the AAC, DPC, HYB scores, the outcome of the predictor clearly shows that amino acid sequences belong to type-I microbial rhodopsins proteins. This indirectly indicates the information that those rhodopsin proteins belongs to Haloarcheal rhodopsins or Prokaryotic rhodopsins. Among *Haloarchaeal* rhodopsins, we can also predict the above amino acid sequence belongs to proton pumping or non-proton pumping rhodopsin proteins

### Data set preparation

The most-reported proton-pumping rhodopsins are in NCBI databases as bacteriorhodopsin, actinorhodopsin, proteorhodopsin and xanthorhodopsin. We retrieved the various microbial rhodopsin sequences from the uniport database using the protein's keyword (https://www.uniprot.org/). The sequences labelled “fragments,” “isoforms,” “potentials,” “similarity,” or “probables” were removed. Furthermore, the CD-hit programme was used to reduce redundancy with a cutoff of 90% ensuring that no two sequences in the dataset share more than 90 percent of redundancy [29].

The final dataset includes 366, 139, 23, 191, 16, and 167 sequences from bacteriorhodopsin, actinorhodopsin, halorhodopsin, proteorhodopsin, sensoryrhodopsin and xanthorhodopsin respectively, the complete datasets are available publicly at the following link (https://bioinfo.imtech.res.in/servers/rhodopred/download.php)

### Amino acid composition

AAC was initially computed by dividing the fraction of each amino acid in a protein by the total number of amino acids. The AAC profile generated a final output of 20. The DPC was calculated by dividing the fraction of each dipeptide in a protein by the total number of dipeptides with a pattern length of 400(20X20) [30].

The percentage of each amino acid present in a protein is referred to as its amino acid composition (AAC).

Data must be encoded into vectors in order for the SVM light to run. The following equation was used to determine the percentage of each of the 20 naturally occurring amino acids:

1 $${\text{Fraction}}\;{\text{of}}\;{\text{amino}}\;{\text{acid}}\;({\text{i}}) = \frac{{{\text{Total}}\;{\text{number}}\;{\text{of}}\;{\text{amino}}\;{\text{acid}}\;({\text{i}})}}{{{\text{Total}}\;{\text{number}}\;{\text{of}}\;{\text{amino}}\;{\text{acids}}\;{\text{in}}\;{\text{protein}}}}$$

### Dipeptide composition

DPC was calculated in the same way, using a vector with a fixed length of 400 (20 × 20) dimensions. The following equation was used to determine the fraction of each dipeptide composition [31]:

2 $${\text{Fraction}}\;{\text{of}}\;{\text{dipeptide}}\;({\text{i}}) = \frac{{{\text{Total}}\;{\text{number}}\;{\text{of}}\;{\text{dipep}}\;({\text{i}})}}{{{\text{Total}}\;{\text{number}}\;{\text{of}}\;{\text{all}}\;{\text{possible}}\;{\text{dipeptides}}}}$$

### Hybrid approach

To increase the prediction accuracy the HYB approach was developed. A prediction model that combines two or more profiles is known as a hybrid model. This study used 420 vector lengths to create hybrid models that included AAC and DPC. The GPSR 1.0 package’s *col_add* function was used to combine the AAC and DPC profiles to create a hybrid profile (https://webs.iiitd.edu.in/raghava/gpsr/).

### Support vector

A SVM is a supervised machine learning technique (MLT) used for classification and regression analysis. For SVM implementation, predictive models were developed by converting the various sequence length into fixed length vectors by implementing several sequence properties. We have used SVM^light^ v6.02 to predict the various types of microbial rhodopsin proteins. While the performance was optimized using RBF kernel on diverse g and c values [32].

### Random forest

Random forest (RF) is an ensemble-learning method based on decision tree model having bootstrapping algorithm. Firstly, decision tree was developed from training data sets and the classes of unknown sample is assigned either according to the mode of classes either in the classification or regression based data sets. We have used RF through Waikato Environment for Knowledge Analysis (WEKA) package for developing a prediction model [33].

### Cross validation

We have used 5-fold and 10-fold cross validation method to evaluate the performance of all the module. For 10-fold cross validation, the data set is randomly divided into 10-equally sized sets [34]. From the 10 sets, one set is used for testing while the remaining nine sets are considered for training. This process is repeated ten times and each set will get the chance to be the testing data set. Likewise, in 5-fold cross validation, data set is divided into 5-sets, where 1 set is tested by the model developed on the remaining 4 sets. This process is also iterated 5 times.

### Performance measures

The performance of the predictive models was evaluated by calculating specificity (SP), sensitivity (SN), accuracy (ACC) and Mathew's correlation coefficient (MCC) using the following equations [35]:

3 $$Accuracy\;(ACC) = \frac{TP + TN}{{TP + TN + FP + FN}}$$

4 $$Sensitivity\;(SN) = \frac{TP}{{TP + FN}}$$

5 $$Specificity\;(SP) = \frac{TN}{{TN + FP}}$$

6 $$MCC = \frac{TP \times TN - FP \times FN}{{\sqrt {\left( {TP + FP} \right)\left( {TP + FN} \right)\left( {TN + FP} \right)\left( {TN + FN} \right)} }}$$

### Webserver

Rhodopred webserver is developed using LAMPP software. The front-end was developed using PHP, HTML, CSS, JavaScript and PERL. The backend was linked to the apache server using linux platform. The webserver is freely accessible at https://bioinfo.imtech.res.in/servers/rhodopred. We have also provided the general information of webserver in the “About” section. Rhodopred webserver is a machine learning based classification method for predicting various microbial rhodopsin proteins. Rhodopsin protein modeling was done using support vector machines (SVM) and their classes, viz. actinorhodopsin, bacteriorhodopsin, halorhodopsin, proteorhodopsin, sensoryrhodopsin and xanthorhodopsin. On the home page, the user can paste/upload the protein sequence (fasta or multiple fasta) in the textbox. This will predict the input protein sequence as rhodopsin (YES) or non-rhodopsin (NO) proteins based on SVM score for amino acid composition (AAC), dipeptide composition (DPC) and hybrid (AAC+DPC). Users can also predict rhodopsin protein for each class by selecting each rhodopsin protein in the “Class” section of the webserver. It will also provide score and predict whether the sequence belongs to a particular rhodopsin protein or not.

## Results

**Fig. 1:**
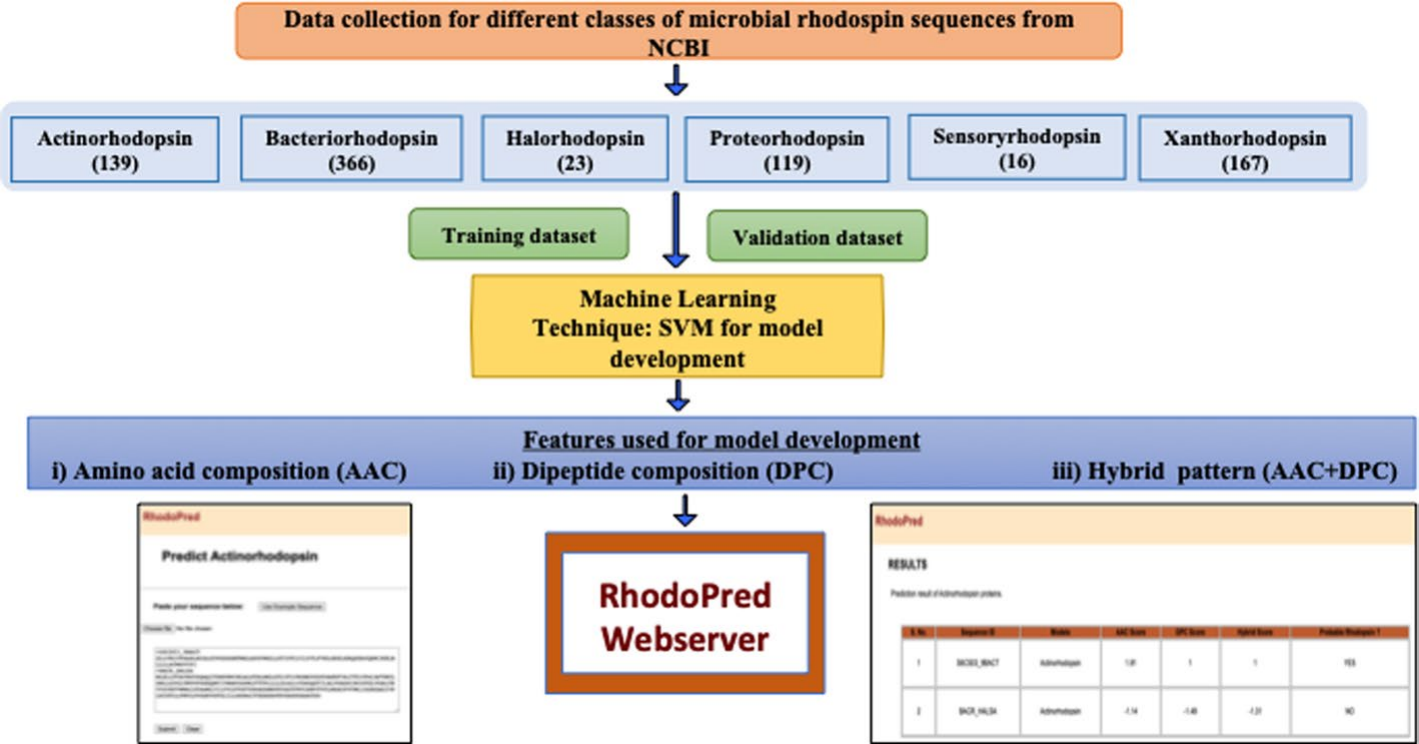
Flow Chart for developing SVM method to predict microbial rhodopsin proteins

Many computational approaches are currently available for predicting diverse functional proteins utilizing a machine learning methodology. This work is concerned with predicting and analyzing various microbial rhodopsins and analysing our recently isolated *Haloarchaeal* strains of whole genome data available in NCBI database (https://www.ncbi.nlm.nih.gov/genome/). The developed SVM approaches were also evaluated against the annotated whole genome sequence of PWS Haloarchaeal isolates. According to our findings, the established approach accurately identifies the rhodopsin sequences and various types of Type-I microbial rhodopsins (Fig. 1).

### Analyze the aminoacid profile of microbial rhodopsin

**Fig. 2:**
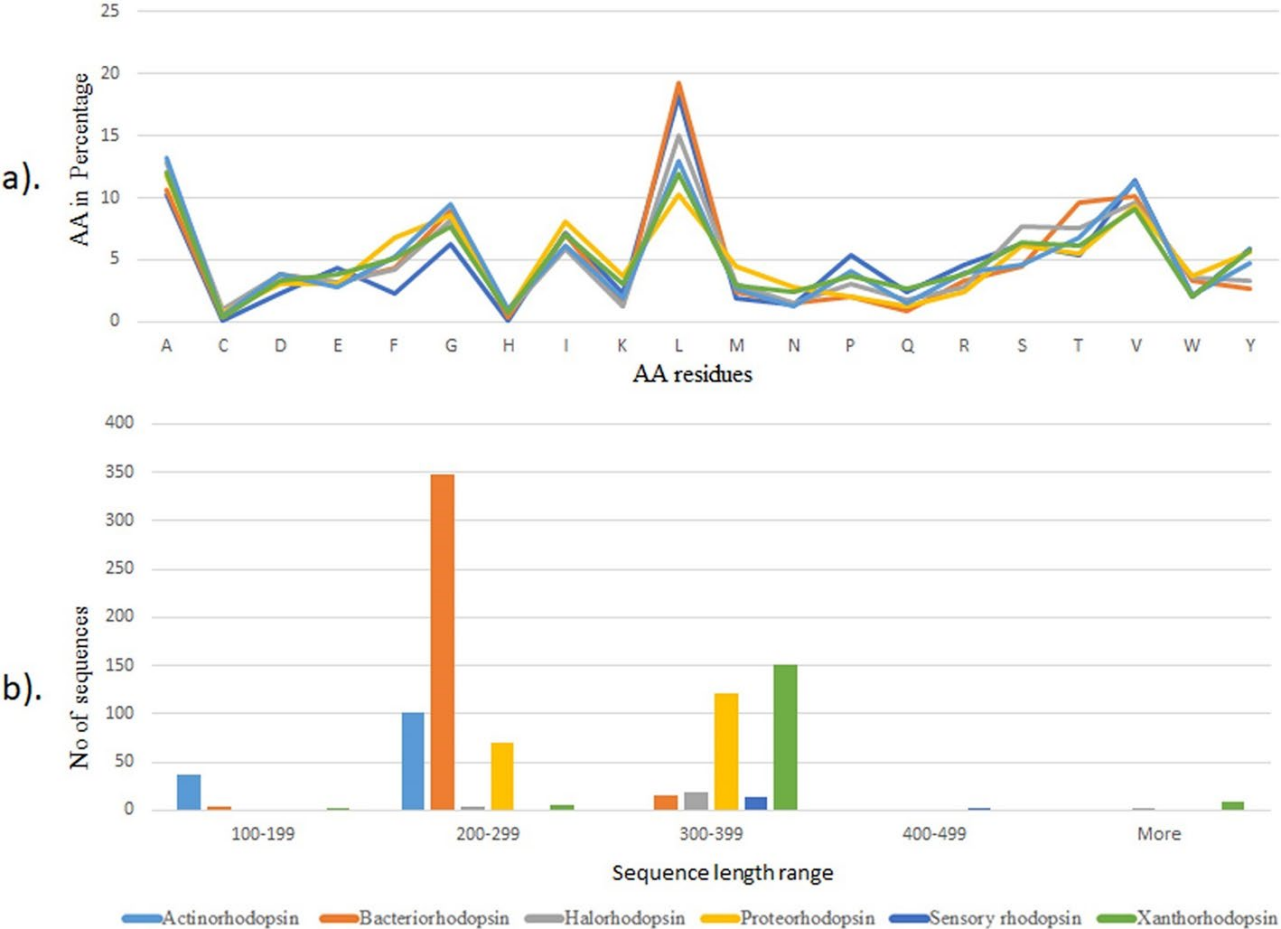
**a** Amino acid distribution chart of Ion Pumping Type-1 Microbial rhodopsin. **b** Aminoacid Sequence length distribution profile of Type I Microbial rhodopsin

The average amino acids for various rhodopsin proteins were computed, and residues “L” and “A” are present in more than 10% of all rhodopsins. A high abundance of these non-polar amino acids like Leucine and Alanine are signature amino acids for integral membrane proteins like Microbial Type-I rhodopsins. Compared to other rhodopsins, bacteriorhodopsin and sensory rhodopsin make up almost 20% of the total residues “G” and “V” in excess of 5%. The residues “C” and "H" are mostly missing. The remaining residues are found in all rhodopsins in similar amounts. Figure 2a depicts the aminoacid composition of all rhodopsins.

We also computed the sequence length profile of several rhodopsins and found that the majority of the sequences were between 200 and 399 amino acids long. Interestingly, the majority of the bacteriorhodopsin sequences are in the 200–299 ranges. Furthermore, most xanthorhodopsin and Proteo rhodopsin sequences are found in the 300–399 ranges. Other rhodopsins, such as Sensory, Halo, and Actino rhodopsins, are found in various lengths 200–299, 300–399. The details of the results are shown in Fig. 2b.

### Performance of AAC-SVM based classification

The entire classes of rhodopsin performed equally well during 10-fold cross validation. For AAC, the maximum accuracy and MCC has been achieved for actinorhodopsin followed by halorhodopsin, sensoryrhodopsin, xanthorhodopsin, proteorhodopsin, bacteriorhodopsin and overall with 99.88%, 1; 99.75%, 0.95; 99.38%, 0.80; 98.65%, 0.96; 98.27%, 0.95; 98.15%, 0.96 and 97.78%, 0.96 respectively during 10-fold cross validation. These models showed equal performance on independent data set on all classes of rhodopsin as shown in Table 1. Further, rhodopsin classes also performed well during 5-fold cross validation as given in Additional file 2: Fig. S1, Additional file 1: Table S1.

**Table 1:** Performance of SVM based predictive models for different classes of rhodopsin during tenfold cross validation

Class	Datasets	Type	ACC	MCC	AUC	GC
Overall (902p + 902n)	T^812p+812n^	AAC	97.78	0.96	1.00	g:0.001 c:0.1
	V^90p+90n^		98.89	0.98	1.00	
	T^812p+812n^	DPC	97.84	0.96	1.00	g:0.005 c:0.1
	V^90p+90n^		99.44	0.99	1.00	
	T^812p+812n^	HYB	97.6	0.95	1.00	g:0.005 c:0.01
	V^90p+90n^		98.89	0.98	1.00	
Actinorhodopsin (139p + 763n)	T^125p+687n^	AAC	99.88	1	1.00	g:0.01 c:10
	V^14p+76n^		98.89	0.96	1.00	
	T^125p+687n^	DPC	99.88	1	1.00	g:0.01 c:10
	V^14p+76n^		98.89	0.96	1.00	
	T^125p+687n^	Hybrid	99.63	0.99	1.00	g:0.01 c:10
	V^14p+76n^		100	1	1.00	
Bacteriorhodopsin (366p + 536n)	T^330p+482n^	AAC	98.15	0.96	1.00	g:0.01 c:50
	V^36p+54n^		98.89	0.98	1.00	
	T^330p+482n^	DPC	99.75	0.99	1.00	g:0.01 c:50
	V^36p+54n^		98.89	0.98	1.00	
	T^330p+482n^	Hybrid	99.75	0.99	1.00	g:0.01 c:50
	V^36p+54n^		98.89	0.98	1.00	
Halorhodopsin (23p + 879n)	T^21p+791n^	AAC	99.75	0.95	0.97	g:0.01 c:10
	V^2p+88n^		100	1.00	1.00	
	T^21p+791n^	DPC	99.75	0.95	1.00	g:0.01 c:10
	V^2p+88n^		100	1.00	1.00	
	T^21p+791n^	Hybrid	99.75	0.95	1.00	g:0.01 c:5
	V^2p+88n^		100	1.00	1.00	
Proteorhodopsin (191p + 711n)	T^171p+640n^	AAC	98.27	0.95	1.00	g:0.0001 c:50
	V^20p+71n^		98.9	0.97		
	T^171p+640n^	DPC	98.27	0.95	1.00	g:0.001 c:1
	V^20p+71n^		100	1.00	1.00	
	T^171p+640n^	Hybrid	98.77	0.96	1.00	g:0.001 c:1
	V^20p+71n^		98.9	0.97	1.00	
Sensoryrhodopsin (16p + 886n)	T^14p+798n^	AAC	99.38	0.8	0.90	g:0.01 c:5
	V^2p+88n^		98.89	0.7	0.88	
	T^14p+798n^	DPC	99.38	0.81	0.97	g:0.001 c:10
	V^2p+88n^		98.89	0.7	0.99	
	T^14p+798n^	Hybrid	99.51	0.85	0.93	g:0.01 c:1
	V^2p+88n^		98.89	0.7	0.99	
Xanthorhodopsin (167p + 735n)	T^151p+662n^	AAC	98.65	0.96	1.00	g:0.05 c:1
	V^16p+73n^		98.88	0.96	1.00	
	T^151p+662n^	DPC	99.02	0.97	1.00	g:0.01 c:1
	V^16p+73n^		97.75	0.92	1.00	
	T^151p+662n^	Hybrid	99.02	0.97	1.00	g:0.01 c:1
	V^16p+73n^		97.75	0.92	1.00	

ACC, accuracy; MCC, Matthew’s correlation coefficient; AUC, area under curve; AAC, amino acid composition; DPC, dipeptide composition; Hybrid, AAC + DPC

### Performance of DPC-SVM based classification

For DPC, actinorhodopsin achieved the highest accuracy and MCC followed by bacteriorhodopsin, halorhodopsin, sensoryrhodopsin, xanthorhodopsin, proteorhodopsin, and overall with 99.88%, 1; 99.75%, 0.99; 99.75%, 0.95; 99.38%, 0.81; 99.02%, 0.97; 98.27%, 0.95; and 97.84%, 0.96 correspondingly during 10-fold cross validation. Similarly all models performed equally well on independent data set of all classes of rhodopsin (Table 1). Likewise, rhodopsin classes also showed good performance on 5-fold cross validation (Additional file 2: Fig. S1, Additional file 1: Table S1).

### Performance of HYB-SVM based classification

In case of HYB, bacteriorhodopsin got the maximum accuracy and MCC of 99.75% and 0.99 followed by halorhodopsin, actinorhodopsin, sensoryrhodopsin, xanthorhodopsin, proteorhodopsin, and overall with 99.75%, 0.95; 99.63%, 0.99; 99.51%, 0.85; 99.02%, 0.97; 98.77%, 0.96 and 97.60%, 0.95 respectively during 10-fold cross validation. Similalrly, predictive models also performed equally well on the independent data set (Additional file 2: Fig. S1) (Table 1). Likewise, rhodopsin classes also showed good performance on 5-fold cross validation (Additional file 2: Fig. S1, Additional file 1: Table S1).

### Performance of random forest (RF) based classification

Using RF based algorithm for 10-fold cross validation, we achieved the maximum MCC for actinorhodopsin with 0.99 followed by bacteriorhodopsin, overall, xanthorhodopsin, proteorhodopsin, halorhodopsin and sensoryrhodopsin with 0.98, 0.97, 0.94, 0.93, 0.84, and 0.53 respectively for AAC on the training data set. For DPC, actinorhodopsin also has the highest MCC of 1followed by bacteriorhodopsin, overall, xanthorhodopsin, proteorhodopsin, halorhodopsin and sensoryrhodopsin with 0.99, 0.98, 0.95, 0.95, 0.90, and 0.38 respectively. Likewise in HYB approach, actinorhodopsin has the MCC of 1 followed by bacteriorhodopsin, overall, xanthorhodopsin, proteorhodopsin, halorhodopsin and sensoryrhodopsin with 0.99, 0.98, 0.95, 0.95, 0.87, and 0.46 respectively (Additional file 2: Fig. S1, Additional file 1: Table S2). While, the complete result of rhodopsin classes during 5-fold cross validation on RF algorithm is given in Additional file 2: Fig. S1, Additional file 1: Table S3. Further these models showed equal performance on independent data set as shown in Additional file 2: Fig. S1, Additional file 1: Table S4.

### Confusion matrix performance by prediction scoring graphs

**Fig. 3:**
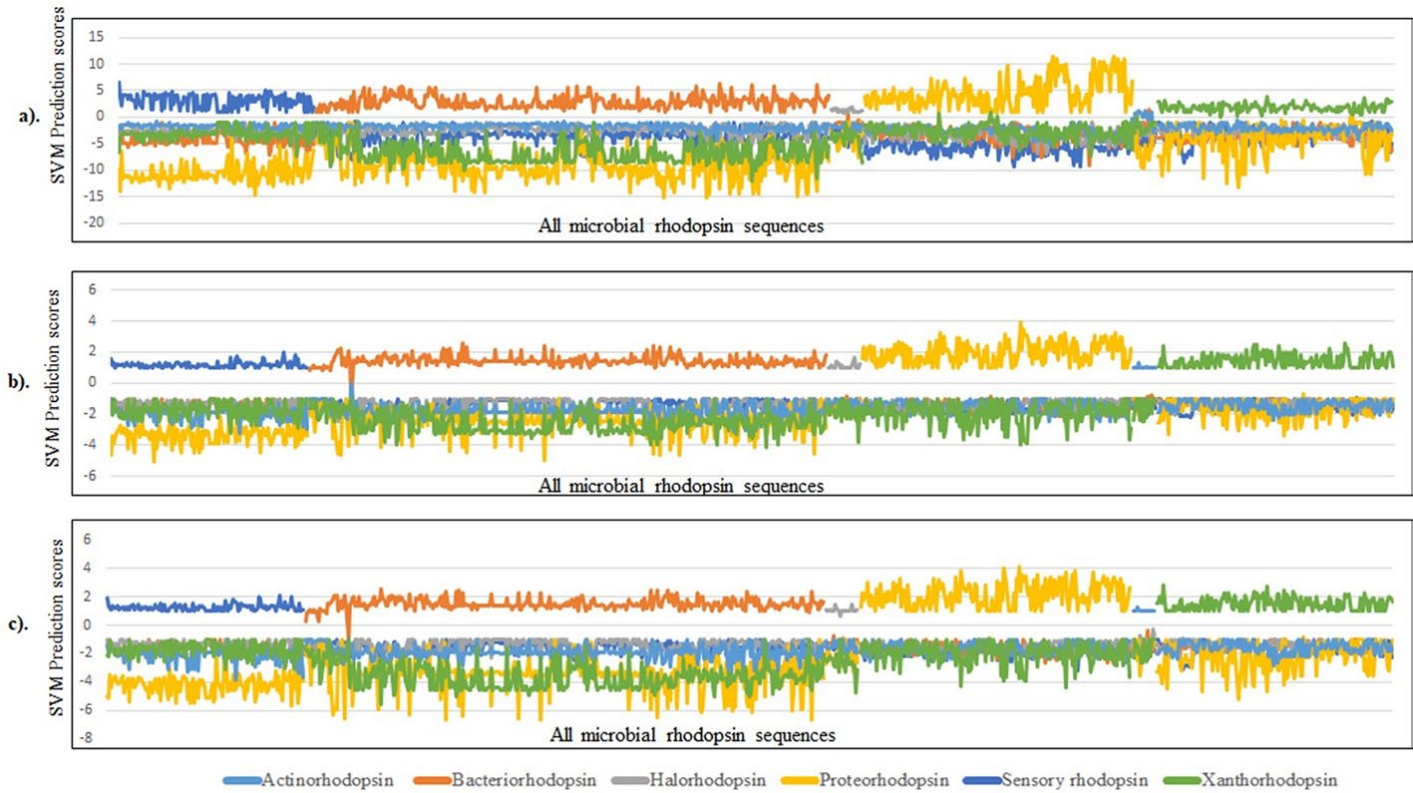
Prediction performances of Confusion matrix **a** Amino acid Composition, **b** Dipeptide Amino acid Composition, **c** Hybrid Composition

The confusion matrix and prediction scoring graphs were also used to assess the performance of SVM modules. The prediction score for each unique sequence studied is depicted in the scoring graph, which shows how a threshold distinguishes the positive set's score from the negative set's score in order to distinguish between positive and negative predictions. However, not all positive or negative sequences can be accurately detected, resulting in false negative and positive predictions. In this analysis, we found that all models such as Amino acid composition, dipeptide composition, the SVM prediction scores for the Amino acid models are found to be positive scores for actinorhodopsin, bacteriorhodopsin, proteorhodopsin and xanthorhodopsin. This confirms the very distinct classification of Type I proton pumping among all Type I microbial rhodopsin. In this amino acid composition model the proton pumping rhodopsins were not confused with the other Type I microbial rhodopsin sequences (Fig. 3a–c).

### BLAST dataset prediction and analysis

To validate of our developed methods microbial rhodopsin protein sequences was extracted from NCBI database to identify BLAST data using our developed models to analyse the performance of the developed models. In this investigation, a total of 500 sequences from each family were employed, with five sequences from our dataset running BLAST and collecting 100 each from a sequence. The output findings demonstrate that on an average 54% of actinorhodopsin BLAST sequences were recognized by its own models, 91% sequences on an average were recognized by bacteriorhodopsin models overlapping with halorhodopsin sequences suggest that bacteriorhodopsin and halorhodopsin sequences were over lapping each other which shows close sequences similarity in rhodopsin amino acid sequences. All models recognise BLAST data sequences. In the other classes, the BLAST sequences were recognised by its own all models as 53.4%, 97.4%, 97.6%, 99.4%, 45.2 %, and 99.6% in actinorhodopsin, (Table 2) bacteriorhodopsin, halorhodopsin, proteorhodopsin, sensory rhodopsin and xanthorhodopsin respectively. Actinorhodopsin and sensory rhodopsin BLAST data prediction percentage scores showing less percentage because of presence of rhodopsin like hypothetical sequences in the NCBI Database. Some sequences were predicted by other models rather than by their own, while a few sequences were recognized by both their own and other class models. Table 3 summarises the findings of this investigation.

**Table 2:** Rhodopred performance on BLAST dataset—overall

Microbial-rhodopsins	No. of seq	BLAST sequences					
		Actinorhodopsin	Bacteriorhodopsin	Halorhodopsin	Proteorhodopsin	Sensory rhodopsin	Xanthorhodopsin
Actinorhodopsin	500	267 (53.4%)					233
Bacteriorhodopsin	500		487(97.4%)	28			
Haloarhodopsin	500			488(97.6%)			
Proteorhodopsin	500				497(99.4%)		
Sensory rhodopsin	500		41	1		226(45.2%)	3
Xanthorhodopsin	500	1					498(99.6%)

**Table 3:** **‘**Rhodopred’ performance on annotated PWS experimental isolates dataset

PWS *Haloarchaeal* isolates	Actino rhodopsin	Bacterio rhodopsin	Halo rhodopsin	Proteo rhodopsin	Sensory rhodopsin	Xantho rhodopsin
> PWS11 Rhodopsin	NO	NO	NO	NO	NO	NO
> PWS12 Rhodopsin	NO	YES	NO	NO	NO	NO
> PWS13 Sensory Rhodopsin2	NO	YES	NO	NO	NO	NO
> PWS5 Cruxrhodopsin Cop3	NO	YES	NO	NO	NO	NO
> PWS5 Sensoryrhodopsin II	NO	NO	NO	NO	YES	NO
> SL3 Rhodopsin 1	NO	NO	NO	NO	YES	NO
> SL3 Rhodopsin 2	NO	YES	NO	NO	NO	NO
> SL3 Sensoryrhodopsin 2	NO	NO	NO	NO	YES	NO
> R1 Bacteriorhodopsin	NO	YES	NO	NO	NO	NO
> R1 Halorhodopsin	NO	NO	YES	NO	NO	NO
> R1 Sensoryrhodopsin I	NO	NO	NO	NO	YES	NO
> R1 Sensoryrhodopsin II	NO	NO	NO	NO	YES	NO
> NRC1 Bacteriorhodopsin bop	NO	YES	NO	NO	NO	NO
> NRC1 Bacteriorhodopsin related protein	NO	NO	NO	NO	NO	NO
> NRC1 Halorhodopsin	NO	NO	YES	NO	NO	NO
> NRC1 Sensoryrhodopsin I	NO	NO	NO	NO	YES	NO
> NRC1 Sensory rhodopsin II	NO	NO	NO	NO	YES	NO

### Rhodopsin genes extraction from annotated whole genome sequence analysis

**Fig. 4:**
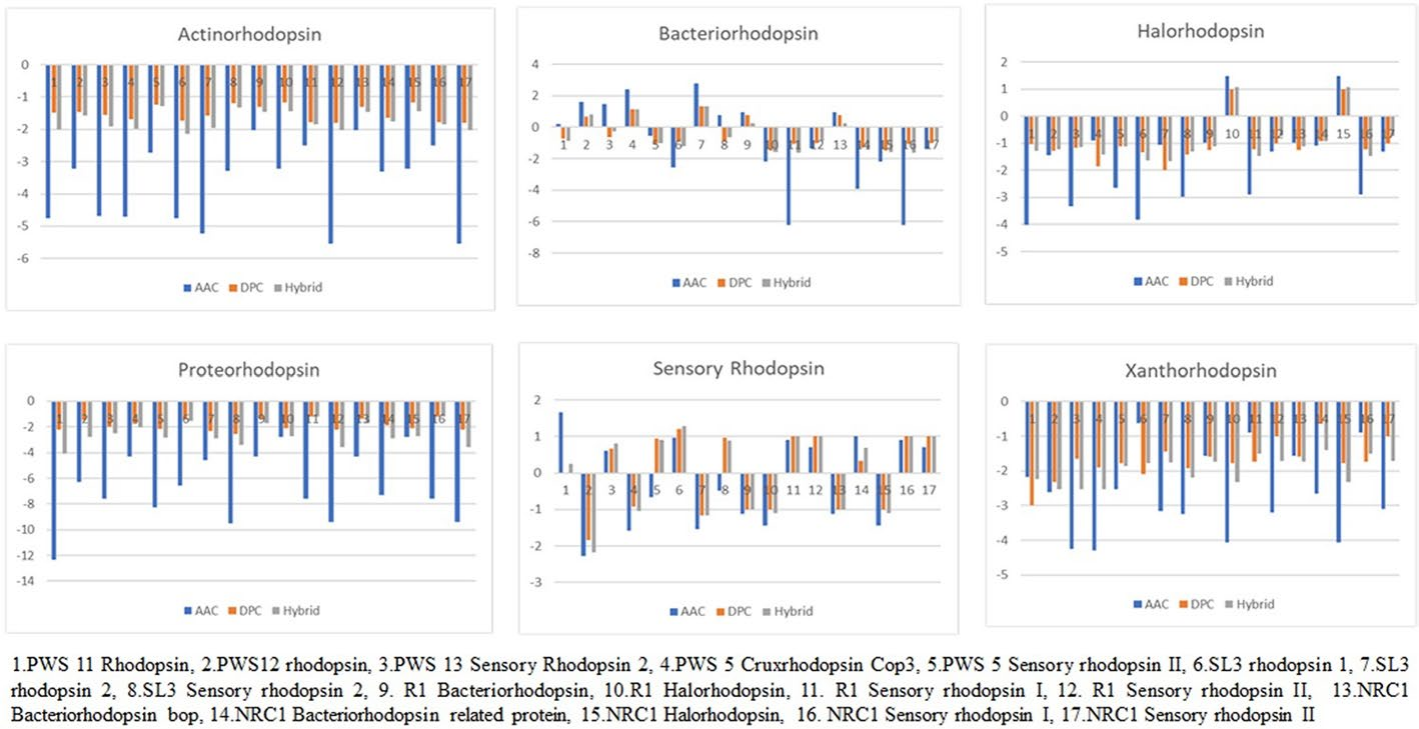
Prediction performance of rhodopsin proteins extracted from annotated *Haloarchaeal* whole genome sequencing

In this study, we used SVM_light to predict the various type-I Ion pumping Microbial Rhodopsin proteins. Whole genome sequencing data of our PWS1,5, SL3 and 11 isolates for identifying Type I microbial rhodopsin genes were analysed from the NCBI genome database (Table 3). Extracting the microbial rhodopsin gene sequences consist of following steps (1) Enter the accession number in the NCBI Database, (2) go to nucleotide sequence, (3) Enter Gen bank number and WGS : WOYG00000000.1, (4) Search rhodopsin in scaffolds. In addition to the microbial Rhodopsin classification, our group recently published and deposited whole-genome sequencing of PWS isolates PWS1,5,11 identified from Pondicherry Solar Salterns. (Pondicherry salterns located in the east coast road of Tamil nadu, India). These extreme haloarcheal isolates (PWS1, PWS5, PWS11) where subjected for whole-genome sequencing yielded 3.39 Mb, 4.0 Mb, 3.67 Mb, and SL3 is reference *Haloarcula* genome. The GC Content was found to be 65.7%, 61.3%, 62.0% and 66.1% for pws1, pws5, SL3, and pws11 respectively. The accession number for PWS1, PWS5, SL3, PWS11 was reported to be WOYG00000000.1, NZ_WOWA00000000.1, LIUF00000000.1, WOWC00000000.1 (Table 4, Fig. 4). The support vector machine classifier clearly distinguished the presence of rhodopsin proteins and Non rhodopsin proteins. In addition the SVM model identified the type of Type-I microbial rhodopsin A single proton pumping Bacteriorhodopsin expression in Halobacterium salianrium requires bop, Blp, brp, crtb, blh genes (Table 5) [36]. Presence of brp, blh, blp, bat, Crtb1 essential genes and structural rhodopsin genes in the reference Halobacterium salianrium NRC1 and R1 whole genome annotated sequence indicates that these two wild type *Halobacterium* strains capable to express milligram per liter scale of native bacteriorhodopsin protein. A total of 17 rhodopsin sequences were employed, with the majority of them recognized as bacteriorhodopsin, halorhodopsin, and sensory rhodopsins as per the whole genome sequence analysis (Fig. 4). Out of 17 microbial rhodopsin sequences extracted from NCBI whole genome database PWS1,5,11 were experimentally verified Haloarchaeal whole genome analysed rhodopsin sequences. Actinorhodopsin, Proteorhodopsin and xanthorhodopsin models showing negative histograms which shows the absence of rhodopsin proteins in the PWS *Haloarchaeal* isolates**.** This indicates that bacteriorhodopsin harboring PWS isolates such as PWS12 rhodopsin, PWS13 Sensory rhodopsin, PWS5 Cruxrhodopsin Cop3, SL3 Rhodopsin2, R1 Bacteriorhodopsin and NRC1 Bacteriorhodopsin bop were identified by all models of bacteriorhodopsin. R1 Halorhodopsin and NRC1 [27, 37] Halorhodopsin were rightly differentiated between other Type-I microbial rhodopsins (Fig. 4). Actinorhodopsin, Proteorhodopsin and Xanthorhodopsin protein models were not identified in the PWS-Isolates confirms our finding these whole genome sequenced rhodopsin sequences originates from extreme haloarchaea not from prokaryotic rhodopsin harboring microorganisms.

**Table 4:** Annotated whole genome sequences of rhodopsin genes from Laboratory Isolated

S. no	*Haloarchaeal Genus*	Actino rhodopsin gene (actR)	Bacterio rhodopsin gene (bop)	Halo rhodopsin gene (hop)	Xantho rhodopsin (Xop)	Proteo rhodopsin (PR)	Sensory rhodopsin (sop)
1	*Halobacterium bacterium salianrium NRC1*	No	Yes	Yes	No	No	Yes
2	*Halobacterium salinarium R1*	No	Yes	Yes	No	No	Yes
3	PWS1 *(Halomicrobium mukohatae)*	No	Yes	No	No	No	No
4	PWS5 (*Haloarcula argentinensis)*	No	Yes	No	No	No	Yes
5	SL3 *(Haloarcula ruprimontori)*	No	Yes	No	No	No	Yes
6	PWS11 *(Halaferax volcanii)*	No	No	No	No	No	No

**Table 5:** Bacteriorhodopsin synthesizing genes analysis from annotated whole genome sequences of Laboratory Isolated PWS *Haloarchaeal* Strains

S. no	Bacteriorhodopsin synthesis genes	Genes expansion	PWS1	PWS5	SL3	PWS11	NRC1	R1
	Present in the *Haloarchaeal* genomes							
1	brp	BR Related protein	No	No	No	No	Yes	Yes
2	blh	brp like protein	No	No	No	No	No	Yes
3	blp	bacterioopsin linked blp	No	No	No	No	No	Yes
4	bop	bacteriorhodopsin protein expressing gene	Yes	Yes	Yes	No	Yes	Yes
5	boa 1/3/4	Homolog to transcription regulator bat	No	No	No	No	Yes	Yes
6	bat	bacerioopsin activator	No	No	No	No	No	Yes
7	boa2	Homolog to transcription regulator bat	No	No	No	No	Yes	Yes
8	CrtB1	Phytoene synthase	No	No	No	No	Yes	Yes
9	CrtB2	Phytoene synthase	No	No	No	No	Yes	Yes

### Rhodopred webserver performance using PWS Isolates rhodopsin sequences

Seventeen microbial rhodopsin sequences retrieved from PWS1,5, SL3 and reference genomes from Haloarchael NRC1 and R1 isolates were fed to rhodopred web server. The rhodopred webserver clearly identifies bacteriorhodopsin and cruxrhodopsin like Bacteriorhodopsin proteins and sensory rhodopsin I and II proteins from PWS1 and PWS5 whole-genome rhodopsin sequences. Absence of bacteriorhodopsin in PWS11 Haloarchael isolates indicates the presence of non-bacteriorhodopsin expressing genes. Bacteriorhodopsin, Halorhodopsin, sensory rhodopsin proteins present in the reference genome of haloarchaeal isolates like *Halobacterium salianirum NRC-1* and R1 confirm that our developed webserver rhodopred accurately predicts the sub types of haloarchaeal rhodopsin proteins. Absence of actinorhodopsins and proteorhodopsin proteins in the respective models of rhodopred webserver indicates the presence of *haloarchaeal* whole genome rhodopsin sequences and absence of Prokaryotes microbial rhodopsins. Among the bacteriorhodopsin proteins identified through rhodopred webserver were further analysed for bacteriorhodopsin synthesizing genes in the NCBI Genome database. Absence of these bacteriorhodopsin genes in the Haloarchaeal genomes will express more red pigmented carotenoids which masks the bacteriorhodopsin protein expression in PWS1,5, SL3 isolates*.*

## Discussion

In halophilic archaea, rhodopsin is a retinal binding protein that provides light-sensitive ion transport and sensory function. Marine and Prokaryotic organisms. It is difficult to express the rhodopsin proteins by culturable methods when all the bacteriorhodopsin synthesizing genes were absent in the genome [38]. The culturable methods for wild type and recombinant rhodopsin protein expression will be expensive and time consuming. Therefore, low-cost computational methods are required to identify the microbial rhodopsins proteins and their related subclasses. This study established a very reliable approach for recognizing several Ion pumping Type-I microbial rhodopsins. The first step is to predict Type-I Microbial rhodopsin and non-Type-I Microbial rhodopsin. The second step is to classify Type-I microbial rhodopsin classifications, such as actinorhodopsin, bacteriorhodopsin, haloarhodopsin, proteorhodopsin, sensory rhodopsin, and xanthorhodopsin. The overall prediction accuracy was achieved above 95% in all approaches except AAC, DPC and Hybrid approaches of actinorhodopsin and sensory rhodopsin. According to the results of the BLAST dataset, the developed methods are performing well in all approaches identifying microbial rhodopsins. In the confusion matrix analysis, the 233 sequences of actinothodopsin were identified by xanthorhodopsin, the results suggest that these two proteins sequences may have a close similarity or it may have an evolutionary relationship with one another. Also the results suggest that some sensory rhodopsin sequences have been identified as bacteriorhodopsin. Overall, according to BLAST data, the related sequences were not identified by the own class models, rather identified by other class protein models. As a result, when running, BLAST is unable to recognize the proper sequences; instead, it retrieves comparable proteins that are not the genuine proteins. So our developed method is successfully identifies the different types of Type-I microbial rhodopsins**.** SVM light and Rhodopred webserver based prediction accurately identifies the Type-I microbial rhodopsin protein sequences from annotated whole genome rhodopsin sequences.

We developed a very accurate method, for identifying various microbial rhodopsins using SVM light and rhodopred webserver with different amino acid approaches. As a result, all the developed models accurately detect the different subtypes of Type-I microbial rhodopsin. All our findings indicate that it is better than the BLAST search in identifying microbial rhodopsin, because the BLAST search did not accurately extract the genuine rhodopsin proteins and instead collected other than microbial rhodopsin. We anticipate that this work will aid researchers in finding new or undiscovered microbial rhodopsins having Ion pumping properties. These models accurately predicted the sub type of Type-I Microbial rhodopsin. The general blast search of microbial rhodopsin brings non specific microbial rhodopsin proteins in large numbers. Reference Halobacterium salinarium NRC1, R1 whole genome annotated data indicates the presence of Bacteriorhodopsin, Halorhodopsin, sensory rhodopsin I, II like genes in the genome [39]. Single bacteriorhodopsin protein in the NRC1 and R1 Halobacterium salinarium consist of bacteriorhodopsin structural and supporting genes like bop, brp, bat, blp, and Ctb1 [40]. Among these five genes expect bop gene four supporting genes were absent in the PWS1,5, SL3 isolates. Further it will explores the possibility for the recombinant rhodopsin protein expression in *E-coli* in functional form by adding all trans retinal chromophore invitro. Our group has recently published our findings on Initial 17 amino acids near the N-terminal rhodopsin sequences helps in the proper expression and folding of proton pumping rhodopsin [41]. Another published report on recombinant PWS-5 BR protein was expressed in *E. coli* with light driven proto pumping property by adding all trans retinal invitro [42]. This is the first detailed studied of Support vector machine based Proton pumping the recombinant bacteriorhodopsin protein expression by fishing it out bop gene using specific primers from these PWS isolates by choosing proper vector and host to demonstrate the light driven proton pumping property [43]. The From these two reported research work from our group and our current developed models by SVM light and Rhodopred webserver would be useful for designing rhodopsin genes primers for heterologous expression of rhodopsin proteins in *E-coli* and other host system for Optogentics and Microbial rhodopsin applications.

APC, DPC and HYB performance were good in recognizing the rhodopsin related proteins. We observed that the developed all approaches were equal performance on the independent dataset. The complete analysis results are shown in the Additional file 2: Fig. S1. The similar performance were observed in 10 and 5-fold cross validation. The SVM and random forest techniques performance were also similar in identification of microbial rhodopsins. Since there is no webserver or methods available for microbial rhodopsin, hence we cannot compare the performance with any other methods.

## Conclusion

There is no separate method is available for predicting the various microbial rhodopsin. A method has been developed (Rhodopred) which accurately identify the rhodopsins. This method is developed with 10-fold and fivefold cross-validation techniques with the approaches of AAC, DPC and HYB. All the developed models are validated with the known and the unknown datasets. We also interested to use a deep learning method for our future studies [44–46]. The developed method will be useful for researches working on microbial rhodopsin proteins.

## Supplementary Information

**Additional file 1.** Performance results of SVM model in 5-fold cross validation.

**Additional file 2.** Performance of SVM models in 10-fold and 5-fold.

## Data Availability

The datasets were generated and analyzed for this study, and it is available publicly at the following link, https://bioinfo.imtech.res.in/servers/rhodopred/download.php. The individual class datasets are also available at the following links, https://bioinfo.imtech.res.in/servers/rhodopred/dataset/Actinorhodopsin.txt, https://bioinfo.imtech.res.in/servers/rhodopred/dataset/Bacteriorhodopsin.txt, https://bioinfo.imtech.res.in/servers/rhodopred/dataset/Halorhodopsin.txt, https://bioinfo.imtech.res.in/servers/rhodopred/dataset/Proteorhodopsin.txt, https://bioinfo.imtech.res.in/servers/rhodopred/dataset/Sensory-rhodopsin.txt, https://bioinfo.imtech.res.in/servers/rhodopred/dataset/Xanthorhodopsin.txt. Webserver http://bioinfo.imtech.res.in/servers/rhodopred, Databases/weblinks, https://www.haloweb.org/, https://www.ncbi.nlm.nih.gov/genome/, https://www.uniprot.org/. The NCBI Nucleotide accession number for PWS1, PWS5, SL3 and PWS11 is WOYG00000000.1, NZ_WOWA00000000.1, LIUF00000000.1, and WOWC00000000.1 respectively. Using these accession numbers in the NCBI Nucleotide Database “rhodopsin sequences” were extracted using below NCBI database weblink. PWS1 whole genome sequence weblink: (use “Open Hyperlink” to see the below weblink) https://www.ncbi.nlm.nih.gov/nuccore/WOYG00000000.1, https://www.ncbi.nlm.nih.gov/Traces/wgs/WOYG01?display=contigs. PWS5 whole genome sequence weblink: https://www.ncbi.nlm.nih.gov/nuccore/NZ_WOWA00000000.1, https://www.ncbi.nlm.nih.gov/Traces/wgs/WOWA01?display=contigs. SLR 3 whole genome sequence weblink: https://www.ncbi.nlm.nih.gov/nuccore/LIUF00000000.1, https://www.ncbi.nlm.nih.gov/Traces/wgs/LIUF01?display=contigs. PWS11 whole genome sequence weblink: https://www.ncbi.nlm.nih.gov/nuccore/WOWC00000000.1, https://www.ncbi.nlm.nih.gov/Traces/wgs/WOWC01?display=contigs.
