# Supplementary material for: Ion-pumping microbial rhodopsin protein classification by machine learning approach
Source: BMC Bioinformatics. 2023 Jan 27;24:29. doi: 10.1186/s12859-023-05138-x (PMC9881276; doi:10.1186/s12859-023-05138-x)
Supplement: Supplementary file 1 — Additional file 1. Performance results of SVM model in 5-fold cross validation. [file 12859_2023_5138_MOESM1_ESM.pdf]

Supplementary Table S1. Performance of SVM based predictive models for different classes of rhodopsin during 5-fold cross validation

Class	Datasets	Type	ACC	MCC	AUC	GC
Overall (902p+902n)	$T^{812p+812n}$	AAC	98.65	0.97	1	g:0.01 c:1
	$V^{90p+90n}$		100	1	1	
	$T^{812p+812n}$	DPC	98.83	0.98	1	g:0.005 c:1
	$V^{90p+90n}$		100	1	1	
	$T^{812p+812n}$	HYB	99.14	0.98	1	g:0.001 c:10
	$V^{90p+90n}$		100	1	1	
Actinorhodopsin (139p+763n)	$T^{125p+687n}$	AAC	99.88	1	1	g:0.01 c:10
	$V^{14p+76n}$		98.89	0.96	1	
	$T^{125p+687n}$	DPC	100	1	1	g:0.01 c:10
	$V^{14p+76n}$		100	1	1	
	$T^{125p+687n}$	HYB	100	1	1	g:0.01 c:10
	$V^{14p+76n}$		100	1	1	
Bacteriorhodopsin (366p+536n)	$T^{330p+482n}$	AAC	98.28	0.96	1	g:0.01 c:10
	$V^{36p+54n}$		98.89	0.98	1	
	$T^{330p+482n}$	DPC	99.75	0.99	1	g:0.01 c:50
	$V^{36p+54n}$		98.89	0.98	1	

	$T^{330p+482n}$	HYB	99.75	0.99	1	g:0.01 c:10
	$V^{36p+54n}$		98.89	0.98	1	
Halorhodopsin (23p+879n)	$T^{21p+791n}$	AAC	99.75	0.95	1	g:0.01 c:10
	V^{2p+88n}		100	1	1	
	$T^{21p+791n}$	DPC	99.88	0.98	1	g:0.01 c:5
	V^{2p+88n}		100	1	1	
	$T^{21p+791n}$	HYB	99.88	0.98	1	g:0.01 c:5
	V^{2p+88n}		100	1	1	
Proteorhodopsin (191p+711n)	$T^{171p+640n}$	AAC	97.78	0.94	1	g:0.005 c:1
	$V^{20p+71n}$		98.9	0.97	1	
	$T^{171p+640n}$	DPC	98.15	0.95	1	g:0.01 c:5
	$V^{20p+71n}$		100	1	1	
	$T^{171p+640n}$	HYB	98.52	0.96	1	g:0.001 c:1
	$V^{20p+71n}$		98.9	0.97	1	
Sensoryrhodopsin (16p+886n)	$T^{14p+798n}$	AAC	99.38	0.8	0.89	g:0.005 c:10
	V^{2p+88n}		98.89	0.7	0.86	
	$T^{14p+798n}$	DPC	99.51	0.85	0.97	g:0.005 c:5
	V^{2p+88n}		98.89	0.7	0.99	

	$T^{14p+798n}$	HYB	99.51	0.85	0.93	g:0.01 c:1
	V^{2p+88n}		98.89	0.7	0.99	
Xanthorhodopsin (167p+735n)	$T^{151p+662n}$	AAC	98.52	0.95	1	g:0.05 c:1
	$V^{16p+73n}$		98.88	0.96	1	
	$T^{151p+662n}$	DPC	99.02	0.97	1	g:0.01 c:1
	$V^{16p+73n}$		97.75	0.92	1	
	$T^{151p+662n}$	HYB	99.02	0.97	1	g:0.01 c:1
	$V^{16p+73n}$		97.75	0.92	1	

ACC, Accuracy; MCC, Matthew's Correlation Coefficient; AUC, Area Under Curve; AAC, Amino Acid Composition; DPC, Dipeptide Composition; Hybrid, AAC+DPC;

Supplementary Table S2. Performance of Random Forest based predictive models for different classes of rhodopsin during 10-fold cross validation

**Class**	**Datasets**	**Type**	**Precision**	**Recall**	**MCC**	**ROC**
Actinorhodopsin (139p+763n)	$T^{125p+687n}$	AAC	1.00	1.00	0.99	1.00
		DPC	1.00	1.00	1.00	1.00
		HYB	1.00	1.00	1.00	1.00
Bacteriorhodopsin (366p+536n)	$T^{330p+482n}$	AAC	0.99	0.99	0.98	1.00
		DPC	0.99	0.99	0.99	1.00
		HYB	1.00	1.00	0.99	1.00
Halorhodopsin (23p+879n)	$T^{21p+791n}$	AAC	0.99	0.99	0.84	1.00
		DPC	1.00	1.00	0.90	1.00
		HYB	0.99	0.99	0.87	1.00
Proteorhodopsin (191p+711n)	$T^{171p+640n}$	AAC	0.98	0.98	0.93	1.00
		DPC	0.98	0.98	0.95	1.00
		Hybrid	0.98	0.98	0.95	1.00

Sensoryrhodopsin (16p+886n)	$T^{14p+798n}$	AAC	0.99	0.99	0.53	0.94
		DPC	0.99	0.99	0.38	1.00
		HYB	0.99	0.99	0.46	1.00
Xanthorhodopsin (167p+735n)	$T^{151p+662n}$	AAC	0.98	0.98	0.94	1.00
		DPC	0.99	0.99	0.95	1.00
		Hybrid	0.99	0.99	0.95	1.00
Overall (902p+902n)	$T^{812p+812n}$	AAC	0.99	0.99	0.97	1.00
		DPC	0.99	0.99	0.98	1.00
		Hybrid	0.99	0.99	0.98	1.00

MCC, Matthew's Correlation Coefficient; ROC, Receiver Operating Characteristic; AAC, Amino Acid Composition; DPC, Dipeptide Composition; Hybrid, AAC+DPC;

Supplementary Table S3. Performance of Random Forest based predictive models for different classes of rhodopsin during 5-fold cross validation

**Class**	**Datasets**	**Type**	**Precision**	**Recall**	**MCC**	**ROC**
Actinorhodopsin (139p+763n)	$T^{125p+687n}$	AAC	1.00	1.00	0.99	1.00
		DPC	1.00	1.00	1.00	1.00
		HYB	1.00	1.00	1.00	1.00
Bacteriorhodopsin (366p+536n)	$T^{330p+482n}$	AAC	0.99	0.99	0.97	1.00
		DPC	0.99	0.99	0.99	1.00
		HYB	0.99	0.99	0.99	1.00
Halorhodopsin (23p+879n)	$T^{21p+791n}$	AAC	0.99	0.99	0.78	1.00
		DPC	0.99	0.99	0.81	1.00
		HYB	0.99	0.99	0.84	1.00
Proteorhodopsin (191p+711n)	$T^{171p+640n}$	AAC	0.98	0.98	0.93	1.00
		DPC	0.99	0.99	0.96	1.00
		Hybrid	0.99	0.99	0.96	1.00
Sensoryrhodopsin (16p+886n)	$T^{14p+798n}$	AAC	0.99	0.99	0.46	0.94
		DPC	0.99	0.99	0.46	1.00
		HYB	0.99	0.99	0.46	1.00
Xanthorhodopsin	$T^{151p+662n}$	AAC	0.98	0.98	0.92	1.00

(167p+735n)		DPC	0.99	0.99	0.95	1.00
		Hybrid	0.98	0.98	0.95	1.00
Overall (902p+902n)	$T^{812p+812n}$	AAC	0.99	0.99	0.97	1.00
		DPC	0.99	0.99	0.98	1.00
		Hybrid	0.99	0.99	0.98	1.00

MCC, Matthew’s Correlation Coefficient; ROC, Receiver Operating Characteristic; AAC, Amino Acid Composition; DPC, Dipeptide Composition; Hybrid, AAC+DPC;

Supplementary Table S4. Performance of independent data set using Random Forest based predictive models for different classes of rhodopsin

Class	Datasets	Type	Precision	Recall	MCC	ROC
Actinorhodopsin (139p+763n)	$V^{14p+76n}$	AAC	0.96	0.96	0.82	1.00
		DPC	1.00	1.00	1.00	1.00
		HYB	0.99	0.99	0.96	1.00
Bacteriorhodopsin (366p+536n)	$V^{36p+54n}$	AAC	0.99	0.99	0.98	1.00
		DPC	1.00	1.00	1.00	1.00
		HYB	1.00	1.00	1.00	1.00
Halorhodopsin (23p+879n)	V^{2p+88n}	AAC	1.00	1.00	1.00	1.00
		DPC	1.00	1.00	1.00	1.00
		HYB	1.00	1.00	1.00	1.00
Proteorhodopsin (191p+711n)	$V^{20p+71n}$	AAC	0.98	0.98	0.94	1.00
		DPC	1.00	1.00	1.00	1.00
		Hybrid	1.00	1.00	1.00	1.00
Sensoryrhodopsin (16p+886n)	V^{2p+88n}	AAC	0.99	0.99	0.70	0.99
		DPC	-	0.98	-	1.00
		HYB	-	0.98	-	1.00
Xanthorhodopsin (167p+735n)	$V^{16p+73n}$	AAC	0.97	0.97	0.88	0.99
		DPC	0.98	0.98	0.92	1.00
		Hybrid	0.99	0.99	0.96	1.00
Overall (902p+902n)	$V^{90p+90n}$	AAC	1.00	1.00	1.00	1.00
		DPC	1.00	1.00	1.00	1.00
		Hybrid	1.00	1.00	1.00	1.00
