# Supplementary material for: Ion-pumping microbial rhodopsin protein classification by machine learning approach
Source: BMC Bioinformatics. 2023 Jan 27;24:29. doi: 10.1186/s12859-023-05138-x (PMC9881276; doi:10.1186/s12859-023-05138-x)

Supplementary figure-1. Performance of SVM based predictive models for different classes of rhodopsin during 10-fold and 5-fold cross validation

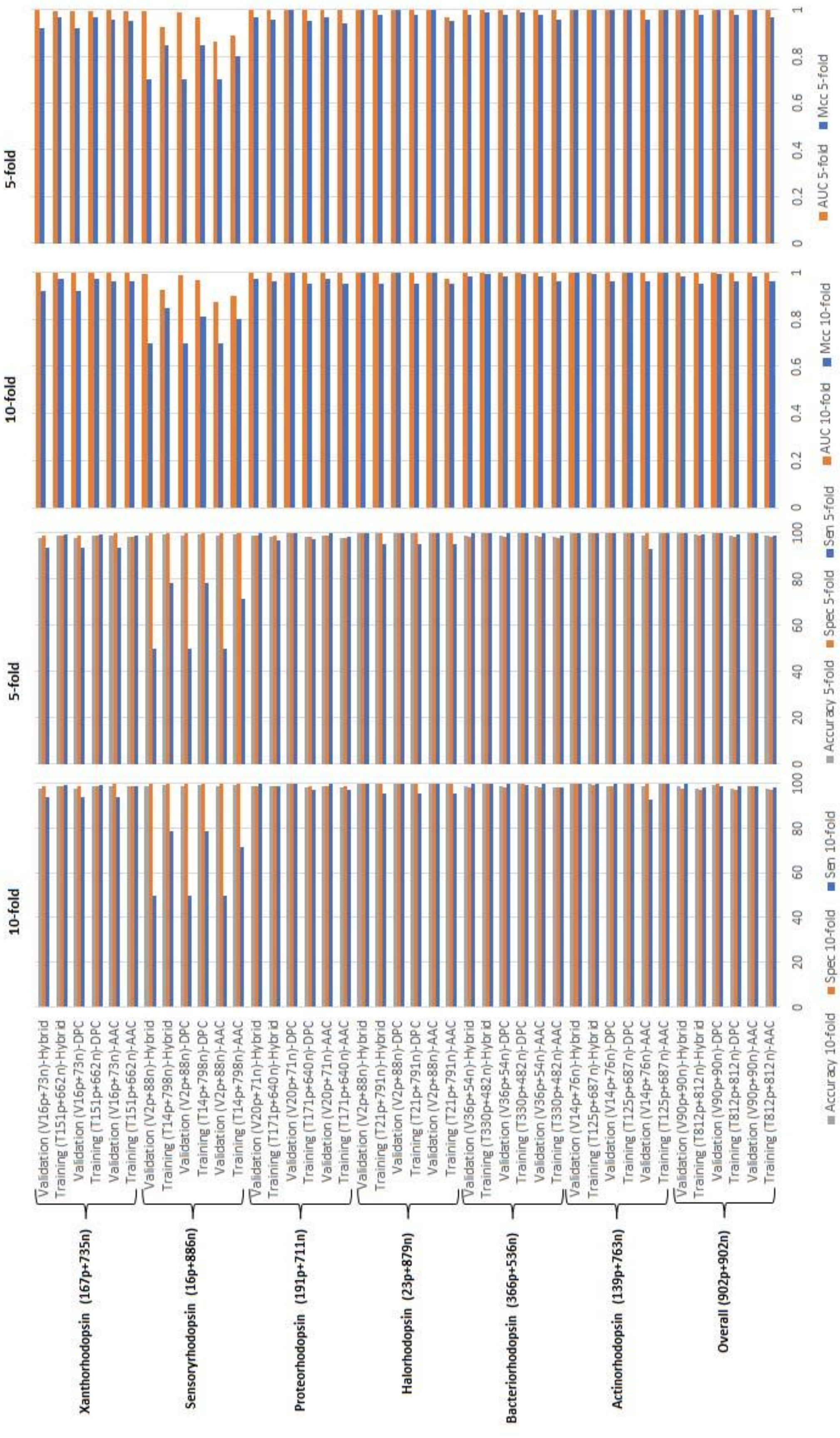

Supplement: Supplementary file 2 — Additional file 2. Performance of SVM models in 10-fold and 5-fold. [file 12859_2023_5138_MOESM2_ESM.pdf]
